# Waiting time interpretations: Complexity and consequences for radiotherapy delays

**DOI:** 10.1016/j.tipsro.2026.100386

**Published:** 2026-02-17

**Authors:** Mruga Gurjar, Jesper Lindberg, Caroline E Olsson

**Affiliations:** a Medical Radiation Sciences, Institute of Clinical Sciences, Sahlgrenska Academy, University of Gothenburg, Sweden; b Department of Biomedical Engineering and Medical Physics, Sahlgrenska University Hospital, Gothenburg, Region Västra Götaland, Sweden; c Regional Cancer Centre West, Western Sweden Healthcare Region, Gothenburg, Sweden

## Abstract

• •Uses clinical scenarios to show effects by different RT waiting time definitions.
• •Highlights the benefits of prioritized start dates − tailored to diagnosis-specific needs.
• •Offers thorough insights to help refine department-specific scheduling strategies.

## Introduction

The number of cancer patients worldwide have increased by 43% in the past decade, leading to a higher demand for radiotherapy (RT) [1]. There are operational challenges in meeting this demand, resulting in long waiting times which may increase the risk of tumour growth [2]. For RT, many studies show evidence of longer delays associating with worse outcomes specifically for head and neck, breast, and bladder cancers [3], [4], [5]. Even with strong healthcare systems, increasing waiting times remains a concern. Previous studies have also noted that there is inconsistency in quantification of waiting times and delays across RT departments [2], [6]. The measurement of waiting times for RT is known to be complex and non-standardized [7], which contributes to substantial variations when comparing queuing statistics and patient timelines. In turn, this makes it challenging to interpret the meaning of acceptable levels for waiting time when using standards of good practice, nationally as well as internationally.

The date of referral to RT and the date of the first treatment fraction is often used to calculate waiting times and to quantify patient numbers on a waiting list. However, a referral may be dated weeks or months before the patient is medically ready to start the treatment. For example, if adjuvant RT is prescribed, the patient may need to recover from other treatments before RT can commence. Some RT departments have therefore added a target date to the referral (due date), when the patient ideally should begin RT given the completion of other treatments. Based on current practices in RT departments, a referral may consequently be assigned several due dates based on the type of treatment. In addition, research on standardizing ideal strategies for waiting time definitions considering both diagnosis and department size perspectives is limited. A recent study based on waiting time management concluded that complex setup requirements for certain treatments directly impact diagnosis-specific waiting times [8]. Another earlier study noted that departments with less than three linear accelerators (LINACs) typically handle few diagnosis groups and require a diagnosis-reliant time management strategy compared to large departments with multiple diagnosis groups [9]. In summary, there are multiple reasons to initiate a scientific analysis of various waiting time definitions and to harmonize comparisons across departments.

The overall aim of this study is to provide departments with an increased awareness about how various waiting time definitions impact delay statistics in RT. We will investigate different strategies to quantify waiting times and explore associated relationships between diagnosis-specific waiting time calculations and different-sized departments.

## Material and methods

To investigate different waiting time definitions, we used scheduling data from a large Swedish RT department during a 12-month period in 2023. To learn about current practices regarding waiting times and delays from a national perspective, all Swedish RT departments delivering photons were asked to share their experience of average delay, waiting time definitions, and periodic follow ups. To investigate effects by varying department characteristics on quantified measures, we utilized data from a national public dataset representing the distribution of total patients across different diagnosis groups for all 16 RT departments delivering photon beam therapy in Sweden.

### Regional RT department

Information on patient and treatment characteristics were retrieved from the oncologic information systems (OIS) − ARIA (Varian Medical Systems, Inc., Palo Alto, CA, U.S.A) at a 13-LINAC large RT department at a major university hospital in Sweden. The dataset included all patients treated during the period from January 2023 to December 2023 and their associated appointment dates between treatment referral to treatment start. In addition, we retrieved information about diagnoses (ICD-10 codes), treatment intent, booking categories, and number of fractions. Details about the investigated department-specific booking categories and diagnosis groups are given in the supplementary material (section 1). Note that treatment intent was retrieved from the OIS booking category data.

In Sweden, patients with a suspected cancer go through so called cancer patient pathways [10]. This means that investigations are standardized and conduced within set time limits to support a speedy process towards treatment initiation. At the studied department, the pathways having RT as a first treatment have pre-booked treatment slots (selected Genitourinary/Gynecologic/Central Nervous System/Head and Neck/Thoracic cancers). Patients eligible for such pre-booked treatment slots are promptly scheduled for treatment upon arrival of the referral and will, therefore, not contribute to the department’s delay statistics. For this reason, patients following cancer patient pathways were excluded from the analysis.

#### Waiting time definitions

**Fig. 1:**
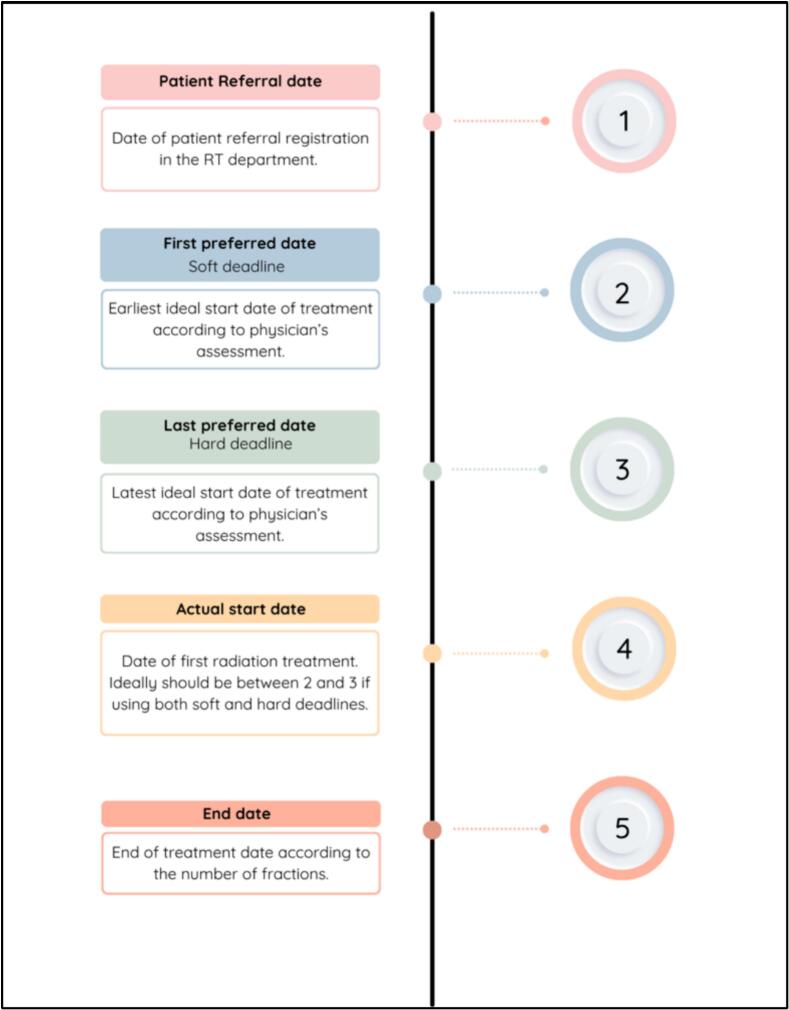
Schematic view of the RT timeline and different types of dates used to calculate delays. Abbreviations: RT: radiotherapy.

Waiting times were computed using four time points: referral date, first preferred date (soft deadline), last preferred date (hard deadline), and treatment start date. Fig. 1 shows a schematic view of how these arise in the overall RT timeline. For the analysis, we used three references, referral date to RT start, soft deadline to RT start and hard deadline to RT start.

We identified diagnosis groups with the highest number of patients and their three most frequent booking categories. For each reference time point, we compared the number of patients who ‘started before’, ‘started on’ and ‘started after’ the date in question by diagnosis group and booking category. A descriptive approach was employed to report the distribution of delays for each group by waiting time definition and with a delay defined as total days exceeding the respective reference date. Descriptive statistics included total values, range, median values, and percentage distribution between different booking categories.

To further identify diagnosis-specific patterns, we visualized the data for the investigated waiting time definitions using histograms. These included number of patients who started treatment by week and diagnosis group with respective booking categories to provide insights into overall delay distribution characteristics.

### National perspective

We designed three open-ended questions to capture the national perspective on waiting times and associated practices. Please refer to the supplementary material (section 2) for a full description of the questions. In short, one question was designed to capture department specific scheduling strategies with respect to waiting time definitions, and two questions to understand average delay and frequency of monitoring them as well as the yearly delay patterns and resolution strategies. Each department was initially approached by e-mail in August 2024 and asked if they were willing to share information about their routines for quantifying and handling waiting times. A first reminder was sent out in October 2024 and a final reminder about 2 months later.

For comparisons and analyses, the departments were divided in three major categories to respect data privacy. Small-sized departments with 2 LINACs, medium-sized departments with 3–5 LINACs and large-sized departments with 6 or more LINACs. A comparison table was created to understand the differences and similarities between departments.

#### National dataset

The national public dataset included information about Swedish RT department’s treatment statistics, resources, research, and use of selected treatments [11]. It represents a 12-month period from January 2023 to December 2023 and provided at the time of this study the most recent official documentation on Swedish RT departments’ characteristics. We used the part of the dataset summarizing each departments’ number of LINACs and treated number of patients per diagnosis group. The dataset also included numbers for both curative and palliative treatment intent.

The national data were specifically used to supplement the questionnaire responses and to illustrate how results by different waiting time definitions from one context can be interpreted in another context. From this dataset, we used averaged total numbers by department size for the largest three diagnosis groups and related these to results for the regional dataset. A scaling factor was calculated for each diagnosis group to reflect the relative proportion between diagnosis groups. Numbers were also normalized to get the *estimated* total patient volume by diagnosis group and department size. For the analysis, we assumed that the booking categories within each diagnosis group were comparable nationally. We further visualized these data based on diagnosis groups and waiting time definitions. Detailed information on scaling and normalization is given in the supplementary material (section 3).

## Results

### Regional dataset

The scheduling data used to investigate the three waiting time definitions encompassed a total of 4827 patients including patients from the pre-booked cancer patient pathways. After excluding these cases, the total number of patients used for analysis was 4172 patients. The three largest diagnosis groups were breast cancer, prostate cancer and thoracic cancers comprising 1507 (36%) patients, 1088 (26%) patients, 345 (9%) patients, respectively (Table 1). The remaining six cancer diagnosis groups (central nervous system and brain, gastro-intestinal, genitourinary, gynecology, head and neck, miscellaneous) included a total of 1232 (29%) patients. The two most common booking categories for breast were coordinated treatments and standard treatments (Table 1). For prostate it was fiducial marker treatments and standard treatments and for thorax it was coordinated treatments and post-operative treatments. All three diagnosis groups had the palliative treatment booking category among the three most common.

**Table 1:** *Number of days exceeding the referral date, first preferred date (soft deadline) and last preferred* date (hard deadline) for 4172 patients treated at the studied regional RT department in during 2023. The table includes the total number of patients per diagnosis group and booking category.

Diagnosis group	Breast cancer (n = 1507)												Total Breast		
Booking Category	Co-ordinated (n = 527)			Standard (n = 823)			Palliation (n = 57)			Misc. (n = 100)					
Reference date	Ref.	Soft	Hard	Ref.	Soft	Hard	Ref.	Soft	Hard	Ref.	Soft	Hard	Ref.	Soft	Hard
Started before (nop)	N/A	43	250	N/A	2	609	N/A	2	35	N/A	3	70	N/A	50	964
Median (days)	N/A	−5	−6	N/A	−4	−12	N/A	−3,5	−11	N/A	−3,5	−4	N/A	−4	−8
Range (days)	N/A	−20, −1	−34, −1	N/A	−27, −4	−18, −1	N/A	−4, −3	−35, −3	N/A	−4, −3	−21, −1	N/A	−27, −1	−35, −1
Started on (nop)	0	25	74	0	18	63	0	4	6	1	15	13	1	62	156
Started after (nop)	527	459	203	823	803	151	57	50	15	99	82	17	1506	1394	386
Median (days)	84	8	3	37	17	3	21	9	7	20	4	3	43	10	4
Range (days)	1, 184	1, 72	1, 91	1, 117	1, 91	1, 57	1, 51	1, 37	1, 21	1, 116	1, 16	1, 29	1, 184	1, 91	1, 91
Diagnosis group	Prostate cancer (n = 1088)												Total Prostate		
Booking Category	Fiducial Markers (n = 808)			Standard (n = 73)			Palliation (n = 75)			Misc. (n = 132)					
Reference date	Ref.	Soft	Hard	Ref.	Soft	Hard	Ref.	Soft	Hard	Ref.	Soft	Hard	Ref.	Soft	Hard
Started before (nop)	N/A	66	277	N/A	1	7	N/A	2	40	N/A	5	80	N/A	74	404
Median (days)	N/A	−6,5	−10	N/A	−8	−6	N/A	−6	−4,5	N/A	−3	−6	N/A	−6	−7
Range (days)	N/A	−27, −1	−41, −1	N/A	−8, −8	−16, −1	N/A	−11, −1	−18, −1	N/A	−4, −1	−35, −1	N/A	−27, −1	−41, −1
Started on (nop)	0	18	44	0	5	34	0	1	8	0	13	28	1	37	114
Started after (nop)	808	724	487	73	67	31	75	72	27	132	110	46	1088	973	591
Median (days)	45	20	14	37	15	8	21	8	5		7	8	40	13	9
Range (days)	1, 133	1, 102	1, 88	1, 80	2, 67	1, 56	1,71	1, 57	1, 43	8, 90	1, 30	1, 20	1, 133	1, 102	1, 88
Diagnosis group	Thorax cancer (n = 345)												Total Thorax		
Booking Category	Co-ordinated (n = 33)			Special treatments (n = 86)			Palliation (n = 105)			Misc. (n = 121)					
Reference date	Ref.	Soft	Hard	Ref.	Soft	Hard	Ref.	Soft	Hard	Ref.	Soft	Hard	Ref.	Soft	Hard
Started before (nop)	N/A	1	7	N/A	2	24	N/A	5	60	N/A	4	67	N/A	12	158
Median (days)	N/A	−6	−4	N/A	−7	−5,5	N/A	−4	−6	N/A	−4	−3	N/A	−5	−5
Range (days)	N/A	−6, −6	−13, −1	N/A	−9, −5	–23, −1	N/A	−6, −1	−17, −1	N/A	−7, −3	−19, −1	N/A	−9, −1	–23, −1
Started on (nop)	0	1	3	0	1	8	0	5	4	1	13	25	1	20	40
Started after (nop)	33	30	22	86	83	54	105	95	41	121	104	28	345	312	145
Median (days)	38	14	10	28	16	7	20	10	3	31	8	3	29	12	6
Range (days)	9, 94	1, 35	1, 22	3, 58	2, 36	1, 35	4, 50	1, 36	1, 29	1, 78	1, 31	1, 16	1, 94	1, 36	1, 35
Diagnosis group	Other diagnoses groups (n = 1232)												Total Others		
Booking Category	Post-op (n = 181)			Standard (n = 117)			Palliation (n = 324)			Misc. (n = 610)					
Reference date	Ref.	Soft	Hard	Ref.	Soft	Hard	Ref.	Soft	Hard	Ref.	Soft	Hard	Ref.	Soft	Hard
Started before (nop)	N/A	3	52	N/A	2	54	N/A	13	178	N/A	18	292	N/A	36	576
Median (days)	N/A	−3	−4	N/A	−2	−5	N/A	−4	−6	N/A	−3,5	−5	N/A	−3	−5
Range (days)	N/A	−4, −1	−18, −1	N/A	−4, −1	−25, −1	N/A	−10, −1	−42, −1	N/A	−27, −1	−47, −1	N/A	−27, −1	−47, −1
Started on (nop)	0	4	36	0	1	19	0	18	48	0	74	101	0	97	204
Started after (nop)	181	174	93	117	144	44	324	291	96	610	513	211	1232	1122	444
Median (days)	33	13	5	28	12	7	21	10	7	22	10	7	26	11	7
Range (days)	13, 82	1, 43	1, 32	10, 68	2, 47	1, 33	1, 76	1, 35	1, 29	1, 161	1, 117	1, 99	1, 161	1, 117	1, 99

Abbreviations: Ref. – Referral date, Soft – soft deadline date, Hard – hard deadline date, Misc. – miscellaneous.

**Fig. 2:**
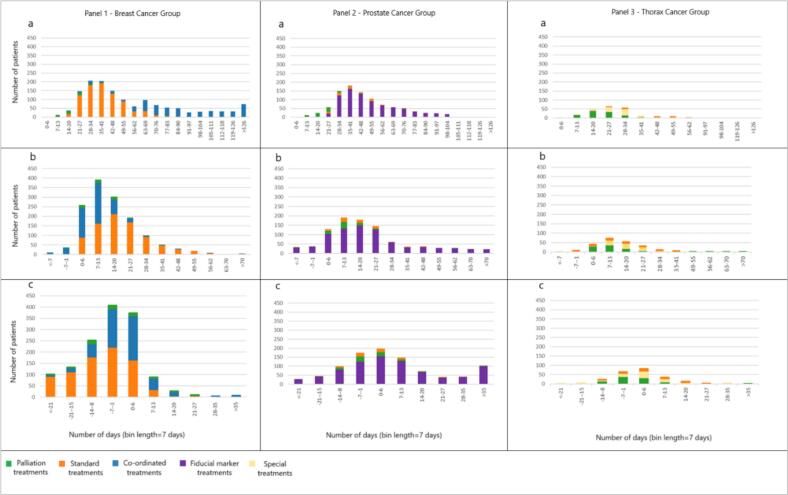
Patient delay distributions by diagnosis for (a) referral date to treatment start, (b) soft deadline to treatment start, and (c) hard deadline to treatment start.

From the standpoint of referral date to RT start, there was limited scope for statistics and meaningful inferences could be drawn only for patients who started after the referral date, with a median delay of 30 days across all groups. Patient distribution for breast, prostate, thorax peaked at 4 ± 1 weeks after the referral date (Fig. 2 – a). Treatment start for palliative treatments was within 2–3 weeks for all diagnosis groups. However, considerable variations were observed for the breast cancer group and coordinated treatments with delays up to 25 weeks (Fig. 2 – Panel 1), for prostate cancer and fiducial marker treatments (Fig. 2, Panel 2), up to 18 weeks. The thorax group showed less variations with a maximum delay for special treatments up to 9 weeks (Fig. 2 – Panel 3).

For the preferred starting dates, altogether, 914 (61%) breast, 330 (30%) prostate and 146 (42%) thorax cases started treatment between the soft and hard deadline with an average delay of 12 days after the soft deadline. Furthermore, 386 (26%) breast, 591 (54%) prostate and 145 (42%) thorax cases started after their hard deadline with an average delay of 7 days (Table 1). A total of 730 (17%) patients started treatment exactly on either the soft or the hard deadline. The distribution of delay with the soft deadline as reference peaked at one week after the deadline for all three diagnosis groups (Fig. 2 – b). For the hard deadline, it peaked at one week before the deadline for breast, and during the same week of the hard deadline for prostate and thorax (Fig. 2 – c). Extensive delays were observed for the prostate cancer group and fiducial marker treatments with 11% of patients starting treatments more than 4 weeks after the hard deadline (Fig. 2 – Panel 2).

### National perspective

**Fig. 3:**
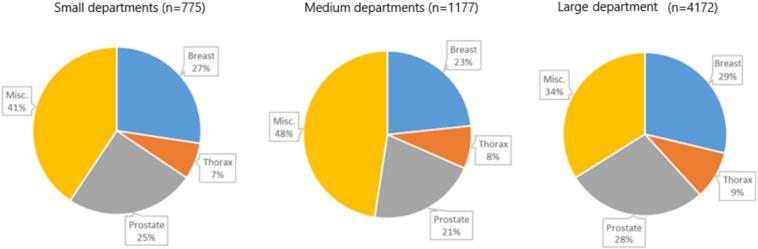
Diagnosis distribution for averaged yearly patient volumes for three of Swedeńs small and eight of the medium RT departments in comparison with the studied large RT department. Abbreviations: Misc − Miscellaneous.

We received responses to the three open-ended questions from 14/16 RT departments (response rate: 87%): 3 from small departments, 8 from medium departments, and 3 from large departments (Table 2). The diagnosis distribution with respect to the three most common groups was similar across all three department sizes, with breast cancers representing the largest group (Fig. 3). Prioritized deadlines to count the delay was reported by 9 departments (64%) while 5 departments (36%) used the referral date. Frequency of monitoring was done regularly on a weekly to quarterly basis at 9 departments. Waiting time definitions differed between departments with only 4 departments (30%) using a time-definitive period from the referral date.

**Table 2:** *Qualitative aspects given responses to three open-ended questions on waiting times and delays* in 2024 from 14 Swedish RT departments.

Department	Dept. 1	Dept. 2	Dept. 3	Dept. 4	Dept. 5	Dept. 6	Dept. 7	Dept. 8	Dept. 9	Dept. 10	Dept. 11	Dept. 12	Dept. 13	Dept. 14
Size	Small	Small	Small	Medium	Medium	Medium	Medium	Medium	Medium	Medium	Medium	Large	Large	Large
Questions														
Waiting time definition	Referral date to actual start date	Preferred date to actual start date	Not specific, delay type-oriented solutions	Preferred date to actual start date	Preferred date to actual start date	Preferred date to actual start date	Preferred date to actual start date	Referral date to actual start date	Referral date to actual start date	Preferred date to actual start date	Referral date to actual start date	Preferred date to actual start date	Referral date to actual start date	2nd preferred date to actual start
Frequency of monitoring	Quarterly	Weekly (twice)	Weekly	Weekly	Not structured	Not structured	Not structured	Quarterly	Not structured	Not structured	Weekly	Weekly	Monthly	Weekly
Treatment schedule timelines	Treatment start within 14 days of referral	Desired starting week to actual start	Not specific	The third earliest available date for treatment start	Referral date to start of treatment	Ideal treatment date to actual treatment start	Preferred date to actual start date	Referral date to start of treatment	Treatment start within 14 days of referral	Not specific	Preparation to start of treatment	Desired starting week to actual start	Referral date to start of treatment	Desired starting week to actual start

Note: Small departments = 2 linacs; medium departments = 3–5 linacs; large departments = 6 or more linacs.

**Fig. 4:**
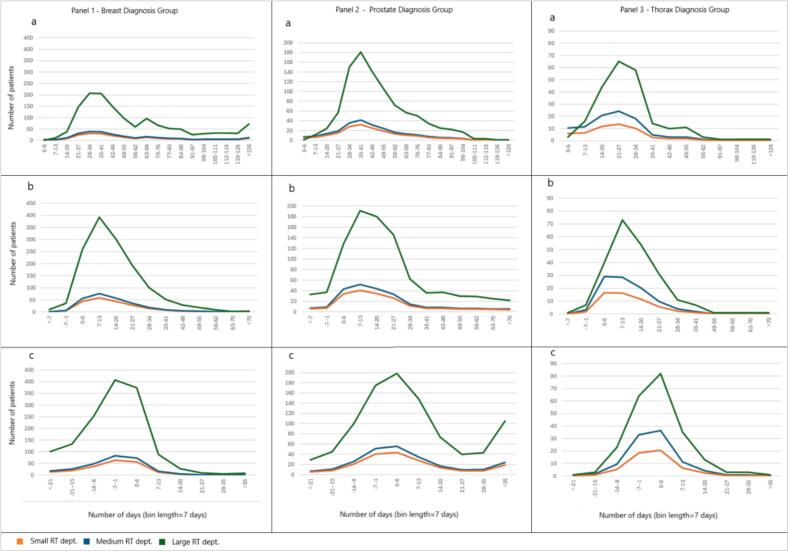
Expected patient delay distributions by cancer diagnosis group assuming correspondence to scaled results from the studied regional RT dataset (large RT department) for (a) referral date to treatment start, (b) soft deadline to treatment start, and (c) hard deadline to treatment start. Abbreviations: Dept – department.

Assuming that the characteristics of each cancer diagnosis group can be regarded as nationally comparable and that Swedish RT departments handle the scheduling of such patients similarly, the expected patient delay distributions by averaged department size and patient volumes can be scaled between departments (Fig. 4). When interpreting this in the context of the national public dataset with referral date as a waiting time reference, this means that regardless of department size, the delay distributions will skew right representing longer delays. If using the soft deadline as reference, around 60% of patients from the three largest diagnosis groups (breast, prostate and thorax) would be expected to start within 14 days after their soft deadline. Correspondingly, if using the hard deadline as reference, approximately 34% of patients of these same diagnosis groups would be expected to start within 7 days after their hard deadline. Additionally, the shape of each distribution can also guide in interpreting delays between waiting time definitions. As an example, translating delays between the three waiting time definitions for the thorax group results in the peak of the referral at 4 weeks moving to 2 weeks for the soft deadline, and to 0 weeks for the hard deadline.

## Discussion and conclusion

Our study highlights the ambiguities of measuring patient delays and investigates three approaches to depict the waiting time in RT. Using detailed scheduling data for 4172 patients treated with RT in 2023 at a large department in Sweden, we found that using the referral as the starting point for waiting time calculations resulted in an overall delay distribution that peaked 4–5 weeks past that date. Using prioritized treatment start dates, the peak was 1–2 weeks after the soft deadline and 1 week prior to the hard deadline. Alongside diagnosis and booking category-specific considerations, analyses of questionnaire responses and a recent public dataset from different-sized RT departments across Sweden showed that there was neither a consistent waiting time definition used across departments, nor was any single delay calculation definition more commonly adopted than others.

The referral date is assigned when a patient is referred for RT. This date is also often entered as the starting point for when the patient formally is registered in the RT department’s OIS. In contrast, the preferred dates rely on professional assessment and are based on clinical judgement [12], [13]. The studied department utilised two prioritized dates when scheduling patients. This strategy had proved to aid operational planning by increasing scheduling flexibility in the past. In our analysis when considering referral date to RT start, calculating median delays per diagnosis group did not account for patient readiness or other external factors that may have intentionally delayed treatment start. These delays are typically observed for patients undergoing curative coordinated treatments. The diagnosis groups most commonly requiring such treatments are breast and prostate cancers which are also two of the largest diagnosis groups worldwide [14]. For palliative treatments in the studied dataset, less than 5% of palliative patients across all diagnosis groups started treatment more than 7 days after the hard deadline. Most began within 1–2 weeks following the soft deadline, indicating a relatively consistent timeline where the referral to RT start serves as a relevant definition of waiting time. In our data, breast cancer patients receiving any type of curative coordinated treatment showed the widest range between referral and RT start dates (1 to 184 days). Yet, according to the prioritized deadline statistics, these longer durations were appropriate and did not necessarily indicate scheduling issues or queues. For instance, out of all breast coordinated treatments, nearly four in ten patients began treatment after the hard deadline. Out of these, only one in ten patients experienced a delay of more than 7 days beyond the hard deadline. For the remaining three booking categories, just under 20% of patients began treatment after the hard deadline out of which a less than 2% of patients experienced a delay of more than 7 days beyond the hard deadline. Delay statistics for the breast cancer group shifted considerably when coordinated cases were handled separately, indicating that a large portion of the delays could be attributed to the complexity and scheduling requirements of coordinated treatments. It also implies that patients undergoing such treatments may require more time to be ready for RT than initially planned − something that for instance can be identified through historical median delays observed for prioritized starting dates.

RT has one of the most elaborate patient timelines due to multiple technical steps including imaging scans, treatment planning, and quality assurance before the start of treatment [13], [15]. Over the years, many scheduling techniques have been explored to efficiently manage such diverse RT patient timelines. However, it has been suggested that a single scheduling strategy may not be suitable for all combinations of diagnoses, booking categories and number of LINACs [16], [17]. In our analysis, we used scheduling data to highlight individual characteristics of diagnoses and treatments and to distinguish regular patients with straight forward timelines from mainstream outliers such as patients from coordinated, post-operative or special treatments exhibiting complex timelines. The studied department utilized both of soft and hard deadlines when scheduling patients. This strategy had proved to aid operational planning by increasing scheduling flexibility (data not shown). Including a buffer to a preferred start date can make it easier to make room for acute patients and to handle expected delays, for instance during longer vacation periods when there will be less staff and fewer treatment slots. During the last decade, more than 20 scheduling models were studied extensively but these rarely accounted for complex diagnosis-specific factors or timelines [18]. A recent framework published by NHS UK provides a structured approach for calculating and reporting large-scale cancer care delays [19]. This framework provides a way to monitor waiting times and compare system level performance metrics nationally, similar to the Swedish cancer patient pathway timelines [10]. However, they do not include detailed perspectives on RT workflow timelines, diagnosis specific recommendations or RT task dependencies. National frameworks, such as the ones in UK and Sweden, would be further strengthened if it incorporated RT workflow-specific characteristics, for example, incorporating patient readiness scores for patients with other treatments prior to RT, similar to the breast cancer example observed in this study. A study published in 2023 used operational research methods to create patient schedules aligning with clinical objectives of curative and palliative patients and resource constraints. One of the objectives used in the mathematical model was minimization of waiting time between referral and treatment start. Our study clearly demonstrated that this metric might not be suitable for all cancer types or treatment pathways with varying periods between referral to treatment start. In addition to the abovementioned situation for breast cancer, we found that prostate cancer patients with fiducial marker treatments consistently showed longer delays compared to other prostate cancer booking categories. However, the cause of these ‘delays’ was because some treatments were less urgent and could tolerate being postponed and, in some cases, were motivated by unexpected patient recovery periods. A suitable way to measure delay for this group of patients could be adjusting the preferred start dates according to the observed average range of the recovery period. Our findings provide opportunities to further improve some of the abovementioned scheduling algorithms with such strategic changes. This would also enable departments worldwide to systematically evaluate their own specific delays rather than focusing solely on an overall one-size-fits-all delay metric.

This study benefits from a real-world high-quality RT dataset directly extracted from the OIS from a large RT department. Furthermore, the use of a national dataset combined with individual responses from the majority of Swedish RT departments strengthened the analysis and revealed waiting time trends for small and medium-sized departments, even without detailed diagnosis- or booking-category-specific information. When addressing delays in RT, it is important to keep in mind that the cancer diagnosis and even the referral to RT are typically determined outside the control of the RT department. In countries outside Sweden, accessing the correct dates from external systems to be transferred into the OIS as a starting point for delay calculations can be difficult. This may especially limit the ability to accurately track waiting time from the time of diagnosis. However, even if work practices vary between regions or countries, our findings indicate that interpreting delays between departments using the scaling of patient volumes, diagnosis and even booking category compositions, can be quite straightforward given insights to the character of available data. A 2012 study in Italy introduced RT prioritization levels based on clinical criteria and time since diagnosis instead of a traditional first come first serve approach [20]. In line with our results, their comparison between approaches showed that the waiting time data differ depending on strategy, in terms of delay in days, distribution of diagnosis and treatment intent. Their study emphasizes that the choice of triage can influence reported waiting times [20]. To sum up, multiple studies have highlighted the importance of standardized definitions and time-to-treatment goals to guide RT departments in managing and comparing waiting times [21], [22], [23]. Observed waiting time trends in this study assume a stable operating environment. Knowing, however, that delays during longer vacation periods (e.g. July-August in Sweden) may be caused by staff shortages [12], staff capacity and resource variations over the year, temporal dynamics need to be studied in more detail to fully understand all aspects of waiting times.

In conclusion, we found that comparisons between diagnosis-specific delay distributions suggest focusing on prioritized treatment start dates for a better understanding of patient timelines, especially for treatments with curative intent and complex timelines where for instance co-ordination with other oncological treatments are needed. However, referral to RT start will still be useful for patients with no other procedures before RT such as single fraction palliative treatments or emergency treatments. Furthermore, applying a booking-category-specific approach may help to better anticipate future scheduling patterns. Overall, our study contributes with novel insights into the delay characteristics in RT and helps refine scheduling strategies given the type of diagnosis treated, different booking categories, and department sizes. Consensus on waiting time definitions and subsequent reporting is currently lacking in the RT community. To this end, we hope that our findings help to lay the groundwork for a strategic and logical approach to quantify and compare various waiting times. Incorporating clear guidance and recommendations for this domain in future national cancer programs is paramount to creating meaningful data that can illustrate both local and global issues.

## Ethics declarations

All data were handled in accordance with ethical and legal requirements. The work in this study was approved by the Regional Ethical Review Board in Gothenburg replaced with the Swedish Ethical Review Authority in 2019 (registration numbers: 841-16, T640-17, 2022-02683-02).

## Data availability

Questionnaire answers and radiotherapy department data contain sensitive patient information and are not publicly available. The national public dataset used in this work is accessible at: https://cancercentrum.se/diagnosbehandling/stralbehandling.724.html.

## Declaration of competing interest

The authors declare that they have no known competing financial interests or personal relationships that could have appeared to influence the work reported in this paper.
