# Supplementary material for: Waiting time interpretations: Complexity and consequences for radiotherapy delays
Source: Tech Innov Patient Support Radiat Oncol. 2026 Feb 17;37:100386. doi: 10.1016/j.tipsro.2026.100386 (PMC12936753; doi:10.1016/j.tipsro.2026.100386)
Supplement: Supplementary Data 1 [file mmc1.docx]

**Supplementary Material**

Section 1: Diagnosis groups and booking categories

Table 1- Diagnosis groups following the structure of the Swedish National Quality Registry with their corresponding diagnoses represented by ICD-10 codes.

| Prostate (1) | Breast (2) | Thorax (4) | Central Nervous System and brain (6) | Genito-  Urinary (8) | Gynecology (8) | Head and Neck  (10) | Gastro-  Intestinal  (15) | Miscellaneous  (46) |
| --- | --- | --- | --- | --- | --- | --- | --- | --- |
| C61 | C50 | C34 | C70 | C60 | C51 | C01 | C15 | C40 |
|  | D05 | C37 | C71 | C62 | C52 | C02 | C16 | C41 |
|  |  | C38 | C72 | C64 | C53 | C03 | C17 | C44 to C47 |
|  |  | C39 | D32 | C65 | C54 | C05 | C18 | C49 |
|  |  |  | D33 | C66 | C55 | C06 | C19 | C73 to C86 |
|  |  |  | D43 | C67 | C56 | C07 | C20 | C88 |
|  |  |  |  | C68 | C57 | C09 | C21 | C91 to C94 |
|  |  |  |  | D41 | D06 | C30 | C22 | C96 |
|  |  |  |  |  |  | C32 | C23 | D02 |
|  |  |  |  |  |  | C69 | C24 | D03 |
|  |  |  |  |  |  |  | C25 | D07 |
|  |  |  |  |  |  |  | C26 | D11 |
|  |  |  |  |  |  |  | C48 | D15 |
|  |  |  |  |  |  |  | D01 | D16 |
|  |  |  |  |  |  |  | D37 | D21 |
|  |  |  |  |  |  |  |  | D24 |
|  |  |  |  |  |  |  |  | D35 to D38 |
|  |  |  |  |  |  |  |  | D44 |
|  |  |  |  |  |  |  |  | D47 |
|  |  |  |  |  |  |  |  | D48 |
|  |  |  |  |  |  |  |  | E05 |
|  |  |  |  |  |  |  |  | H35 |
|  |  |  |  |  |  |  |  | M61 |
|  |  |  |  |  |  |  |  | Q28 |
|  |  |  |  |  |  |  |  | Q85 |

The following are the names of the nine most common booking categories and examples of situations when they are used at the regional RT department in Sweden.

1. Coordinated treatments: RT given together with any other oncologic treatment such as lung cancers undergoing concomitant chemotherapy in combination with RT.
2. Fiducial markers: primarily prostate cancers where fiducial seeds are inserted into the prostate gland used to locate the treatment volume during treatment planning and delivery.
3. Mammillary irradiation: primarily prostate cancers where prophylactic RT is given to prevent hormonally induced growth of glands.
4. Palliation: RT given for any diagnosis with palliative treatment intent.
5. Post-operation: RT given for any diagnosis after surgical removal of tumour
6. Single treatment: primarily palliative treatments where one high-dose fraction is given for pain relief due to bone metastases.
7. Special treatment: RT requiring non-standard handling such as pediatric cancers.
8. Standard appointments: RT requiring no specific coordination, includes routine and uncomplicated treatments for all diagnosis groups.
9. Standard care pathways: treatments with a pre-booked RT workflow which are automatically booked for RT ahead of time. These pathways are specific to each cancer diagnosis.

Section 2: Questions capturing the national perspective on waiting times and associated practices

Open-ended questions sent to RT departments across Sweden (English translation – originally sent in Swedish)

1. **Department specific strategies:** Within your department, how is waiting time defined? Does this encompass the entire duration from referral to the start of treatment, or are there different phases (e.g., from referral to preparatory tasks, and from preparatory tasks to treatment start) that are measured separately?
2. **Delay statistics:** How do you quantify the delay time for patients scheduled for RT? Is this measured from the referral date, the preferred start date, or another specific date? Additionally, do you use a range or a specific cut-off to define delay time from when the treatment actually started?

1. **Frequency of Monitoring:** How frequently do you check for delays in patient scheduling? Are there routine checks or specific protocols followed to identify and address delays? Is monitoring done continuously or at certain time points. Is there a given limit for how many patients that should be scheduled (per week or other time span) to avoid queuing or delays?

Section 3: Scaling and Normalization

Scaling and normalization for small and medium departments

**Scaling** - Departments may have different percentages of diagnosis distributions, a scaling factor was calculated for each diagnosis group based on its proportions across small and medium departments compared to a large department.

**Normalization** – Normalization is applied if there is a need to adjust the values to a common scale across departments, to compare them directly despite differences in size or throughput.

Example calculation – Translating delay results from a large department to a small

Starting with the fact that 386 women with breast cancer had an average delay of 4 days after the hard deadline at the large department (cf. manuscript table 1) and that the yearly total number at the large department is 4172 with 36% being breast cancers. In terms of the small department, how many women would be expected to be delayed to the same reference knowing that the small department treats 775 patients yearly with 27% being breast cancers?

Solution: The large department treats 4172 x 36% = 1507 breast cancers of which 386 starts after the hard deadline (26%).

Scaled value = Percentage breast total small dept./percentage breast total large dept. = 0.27/0.36 = 0.75, i.e. the proportion of breast cancers at the small department is 75% of the proportion of breast cancers at the large department.

Normalized value = total small/total large = 775/4172 = 0.19, i.e. the total number of patients at the small department is 19% of the number of patients at the large department.

Total number of women delayed to the same reference as the large department = 1507 x 0.75 x 0.19 x 0.26 = 55 women, the same as 209 x 0.25 = 55 women.

Table 2 – Averaged values per diagnosis group for small and medium departments (cf. Figure 3). Values for the large department are taken directly from the originally extracted dataset from the regional RT department in Sweden.

| **Dept. size** | **Small department** | | **Medium department** | | **Large department** | |
| --- | --- | --- | --- | --- | --- | --- |
| Diagnosis | Numbers | % | Numbers | % | Numbers | % |
| Breast | 209 | 27% | 271 | 23% | 1507 | 36% |
| Prostate | 194 | 25% | 247 | 21% | 1088 | 26% |
| Thorax | 54 | 7% | 94 | 8% | 345 | 8% |
| Misc. | 318 | 41% | 565 | 48% | 1232 | 30% |
| Total | 775 | 100% | 1777 | 100% | 4172 | 100% |
